# Supplementary material for: Arginine Homopeptide of 11 Residues as a Model of Cell-Penetrating Peptides in the Interaction with Bacterial Membranes
Source: Membranes (Basel). 2022 Nov 24;12(12):1180. doi: 10.3390/membranes12121180 (PMC9788509; doi:10.3390/membranes12121180)
Supplement: Supplementary file 1 [file membranes-12-01180-s001.zip › membranes-2027351-supplementary.pdf]

R11 homopeptide as a model of cell penetrating peptides in the interaction with bacterial membranes

Supplementary material

Table S1. Summary of the main features for Ala scanning peptides.

Substitution position	Sequence	Retention time (min)	Molecular mass	
			Calculated	Observed
**R11**	RRRRRRRRRRR	4.18	1736.1	1736
**A1**	**A**RRRRRRRRRR	4.20	1650.99	1650
**A2**	R**A**RRRRRRRRR	4.41	1650.99	1650
**A3**	RR**A**RRRRRRRR	4.25	1650.99	1650
**A4**	RRR**A**RRRRRRR	4.43	1650.99	1650
**A5**	RRRR**A**RRRRRR	4.21	1650.99	1650
**A6**	RRRRR**A**RRRRR	4.25	1650.99	1650
**A7**	RRRRRR**A**RRRR	4.26	1650.99	1650
**A8**	RRRRRRR**A**RRR	4.23	1650.99	1650
**A9**	RRRRRRRR**A**RR	4.29	1650.99	1650
**A10**	RRRRRRRRR**A**R	4.27	1650.99	1650
**A11**	RRRRRRRRRR**A**	4.27	1650.99	1650

Table S2. Summary of the main features for Pro scanning peptides.

Substitution position	Sequence	Retention time (min)	Molecular mass	
			Calculated	Observed
**R11**	RRRRRRRRRRR	4.18	1736.1	1736
**P1**	**P**RRRRRRRRRR	4.16	1677.13	1676
**P2**	R**P**RRRRRRRRR	4.17	1677.13	1676
**P3**	RR**P**RRRRRRRR	4.14	1677.13	1676
**P4**	RRR**P**RRRRRRR	4.15	1677.13	1676
**P5**	RRRR**P**RRRRRR	4.14	1677.13	1674
**P6**	RRRRR**P**RRRRR	4.13	1677.13	1677
**P7**	RRRRRR**P**RRRR	4.14	1677.13	1677
**P8**	RRRRRRR**P**RRR	4.16	1677.13	1676
**P9**	RRRRRRRR**P**RR	4.19	1677.13	1677
**P10**	RRRRRRRRR**P**R	4.22	1677.13	1676
**P11**	RRRRRRRRRR**P**	4.24	1677.13	1676

Table S3. Summary of the main features for Leu scanning peptides.

Substitution position	Sequence	Retention time (min)	Molecular mass	
			Calculated	Observed
**R11**	RRRRRRRRRRR	4.18	1736.1	1736
**L1**	**L**RRRRRRRRRR	4.27	1693.17	1692
**L2**	R**L**RRRRRRRRR	4.25	1693.17	1692
**L3**	RR**L**RRRRRRRR	4.23	1693.17	1692
**L4**	RRR**L**RRRRRRR	4.18	1693.17	1692
**L5**	RRRR**L**RRRRRR	4.26	1693.17	1692
**L6**	RRRRR**L**RRRRR	4.25	1693.17	1692
**L7**	RRRRRR**L**RRRR	4.23	1693.17	1692
**L8**	RRRRRRR**L**RRR	4.53	1693.17	1692
**L9**	RRRRRRRR**L**RR	4.28	1693.17	1692
**L10**	RRRRRRRRR**L**R	4.34	1693.17	1692
**L11**	RRRRRRRRRR**L**	4.22	1693.17	1692

Table S4. Summary of the main features for tryptophan scanning peptides.

Substitution position	Sequence	Retention time (min)	Molecular mass	
			Calculated	Observed
**R11**	RRRRRRRRRRR	4.18	1736.1	1736
**W1**	**W**RRRRRRRRRR	4.22	1766.23	1764
**W2**	R**W**RRRRRRRRR	4.25	1766.23	1764
**W3**	RR**W**RRRRRRRR	4.27	1766.23	1764
**W4**	RRR**W**RRRRRRR	4.33	1766.23	1764
**W5**	RRRR**W**RRRRRR	4.36	1766.23	1764
**W6**	RRRRR**W**RRRRR	4.34	1766.23	1764
**W7**	RRRRRR**W**RRRR	4.38	1766.23	1764
**W8**	RRRRRRR**W**RRR	4.36	1766.23	1764
**W9**	RRRRRRRR**W**RR	4.37	1766.23	1764
**W10**	RRRRRRRRR**W**R	4.31	1766.23	1764
**W11**	RRRRRRRRRR**W**	4.33	1766.23	1764

Table S5. Two way ANOVA and Tukey's multiple comparison test for the SYTOX green assay. The upper triangular matrix highligthed in yellow corresponds to *S. aureus* and the lower to *E. coli*. Significance level: **** P < 0.000.1. *** P< 0.003; ** P< 0.005; * P< 0.01. ns: not significant.

		S. aureus											
		C-	C+	R11	A3	A10	P1	P6	P7	L2	L3	L9	W7
E. coli	C-		****	****	****	****	****	****	****	****	****	****	****
	C+	****		****	****	****	****	****	****	****	****	****	****
	R11	****	****		ns	****	****	****	*	ns	ns	ns	****
	A3	****	****	****		****	****	****	ns	ns	ns	ns	****
	A10	****	****	ns	****		ns	ns	****	****	****	****	ns
	P1	****	****	****	****	****		ns	****	****	****	****	*
	P6	****	****	ns	****	ns	****		***	****	****	****	****
	P7	****	****	ns	****	ns	****	ns		ns	*	**	****
	L2	****	****	****	****	****	ns	****	****		ns	ns	****
	L3	****	****	****	****	****	****	****	****	****		ns	****
	L9	****	****	****	****	****	ns	****	****	****	****		****
	W7	****	****	****	ns	****	****	****	****	**	*	****	

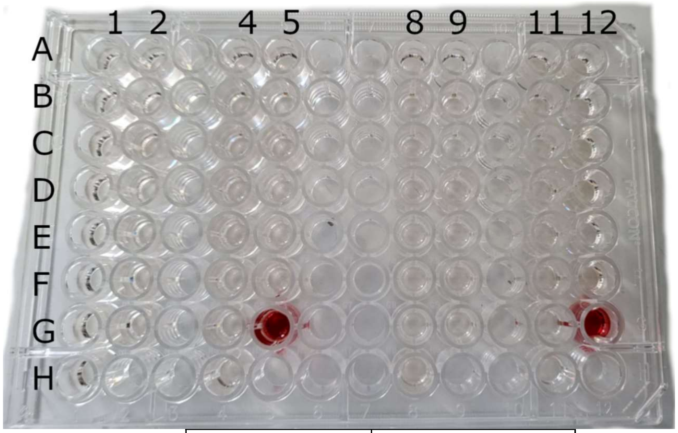

	5 μM		50 μM	
Line	**1, 4**	**2, 5**	**8, 9**	**11, 12**
**A**	R11	L7	R11	L7
**B**	A3	L9	A3	L9
**C**	A1<br>0	L6	A10	L6
**D**	P1	L9	P1	L9
**E**	P6		P6	
**F**	P7	*Bothrops**	P7	*Bothrops**
**G**	L2	0%/100%*<br>*	L2	0%/100%*<br>*
**H**	L3		L3	

**Bothrops* corresponds to the peptide used as control in Sytox and MBC assays.
**0% correspond to PBS 1X in lines 2 and 11, and 100% correspond to triton X-100 in lines 5 and 12.

Figure S1. 96 well plate for the hemolysis assay. Contents of the plate are indicated in the table below the picture.

Table S6. Secondary structure analysis for Ala and Pro scanning performed by CDPro-CONTIN with the SP37A database. Structural classes: H: helix; S: strand; Turn; PP2: Polyproline II. Unrd: Unordered or random. **PPII$_{(Eq2)}$:** percentage of Polyproline II calculated according to equation 2.

2mM PBS																						
Class	R11	A1	A2	A3	A4	A5	A6	A7	A8	A9	A10	A11	P1	P2	P3	P4	P5	P6	P7	P8	P9	P10	P11
H	4	4	4	5	3	5	6	5	3	2	4	6	2	0	5	1	3	2	4	1	30	2	5
S	23	30	23	32	28	25	27	25	22	26	22	28	27	26	22	26	25	23	24	28	0	24	24
Turn	15	14	12	13	11	14	12	14	12	12	12	10	13	12	13	13	12	15	12	13	20	13	13
PPII	12	13	12	13	13	11	13	12	11	11	12	11	12	12	11	13	11	16	11	12	7	12	9
Unrd	46	40	49	37	45	44	42	45	52	50	52	45	47	50	50	47	50	44	49	45	43	49	50
PPII$_{(Eq2)}$	56	48	47	49	46	48	47	51	46	46	47	49	48	45	45	49	44	57	44	40	41	44	49
30% TFE																						
Class	R11	A1	A2	A3	A4	A5	A6	A7	A8	A9	A10	A11	P1	P2	P3	P4	P5	P6	P7	P8	P9	P10	P11
H	6	2	3	4	5	1	3	3	2	2	3	2	4	2	6	5	7	5	7	1	8	3	7
S	21	28	25	32	31	27	30	27	27	27	26	27	20	28	24	22	23	20	23	33	19	27	26
Turn	12	12	12	12	13	12	13	12	12	12	12	13	14	13	12	14	12	14	12	12	13	13	13
PPII	12	12	12	13	13	13	12	12	12	12	12	12	12	13	12	12	11	12	11	15	10	13	11
Unrd	49	46	48	40	39	48	42	47	47	47	47	46	50	45	46	47	47	48	46	39	51	45	44
PPII$_{(Eq2)}$	46	44	45	47	46	47	43	43	45	44	46	45	35	44	37	29	29	33	31	49	27	42	34
E. coli membranes																						
Class	R11	A1	A2	A3	A4	A5	A6	A7	A8	A9	A10	A11	P1	P2	P3	P4	P5	P6	P7	P8	P9	P10	P11
H	3	8	4	1	1	1	6	4	3	6	5	5	1	1	3	1	3	2	2	1	6	1	1
S	24	32	24	27	28	32	24	25	26	34	31	30	24	25	24	25	23	23	24	26	17	24	21
Turn	11	12	15	11	10	11	14	15	12	10	11	11	11	13	14	13	12	13	12	13	13	13	12
PP2	13	15	13	13	14	12	12	12	10	14	9	10	13	14	12	13	13	12	13	13	13	14	13
Unrd	49	33	44	48	47	44	44	44	49	35	44	44	51	47	47	49	50	50	49	47	52	49	53
PPII$_{(Eq2)}$	52	50	61	51	58	50	54	57	49	50	58	49	54	56	54	49	45	43	44	50	41	54	50
DMPG																						
Class	R11	A1	A2	A3	A4	A5	A6	A7	A8	A9	A10	A11	P1	P2	P3	P4	P5	P6	P7	P8	P9	P10	P11
H	0	6	3	1	1	1	0	4	3	5	6	7	1	1	1	2	3	4	3	1	5	3	3
S	21	33	22	28	29	29	29	28	25	31	22	27	22	25	24	26	26	23	23	27	16	21	24
Turn	12	12	11	14	11	11	11	11	11	12	14	13	12	12	12	13	12	13	13	12	12	11	13
PP2	16	15	12	12	13	13	13	13	12	14	12	12	12	13	13	12	11	12	12	13	12	12	11
Unrd	52	33	52	48	46	49	47	45	49	38	45	42	52	50	50	47	48	49	50	47	55	53	50
PPII$_{(Eq2)}$	71	45	46	45	51	51	49	47	47	47	57	50	48	49	49	41	38	36	42	49	43	48	52

Table S7. Secondary structure analysis for Ala and Pro scanning performed by CDPro-CONTIN with the SP37A database. Structural classes: H: helix; S: strand; Turn; PPII: Polyproline II. Unrd: Unordered or random. **PPII$_{(Eq2)}$:** percentage of Polyproline II calculated according to equation 2.

PBS																							
Class	R11	L1	L2	L3	L4	L5	L6	L7	L8	L9	L10	L11	W1	W2	W3	W4	W5	W6	W7	W8	W9	W10	W11
H	4	0	5	0	3	0	4	0	0	3	0	2	0	6	5	0	0	1	1	0	0	2	1
S	23	41	27	31	22	18	25	19	46	24	20	15	25	18	20	58	23	30	21	35	59	8	25
Turn	15	13	16	17	12	13	13	13	11	11	12	14	11	15	17	10	15	14	15	14	12	9	18
PP2	12	18	12	20	13	16	12	17	16	13	18	15	14	9	10	18	15	13	16	18	19	10	18
Unrd	46	29	40	32	49	52	46	52	28	50	51	53	51	53	48	14	47	42	47	34	10	72	38
PPII$_{(Eq2)}$	56	75	71	73	69	75	61	64	65	54	73	70	49	56	51	62	50	47	59	53	49	49	71
30 % TFE																							
Class	R11	L1	L2	L3	L4	L5	L6	L7	L8	L9	L10	L11	W1	W2	W3	W4	W5	W6	W7	W8	W9	W10	W11
H	6	4	1	3	1	6	1	7	8	1	7	6	1	4	1	1	1	2	5	3	1	2	4
S	21	36	29	26	28	36	27	28	26	27	23	23	27	28	27	29	28	29	32	29	29	25	28
Turn	12	16	11	16	12	11	12	14	14	14	16	16	12	15	12	13	12	14	17	14	13	14	16
PP2	12	15	14	11	14	17	13	12	12	13	12	12	14	12	14	14	14	14	14	13	13	13	14
Unrd	49	31	45	44	46	30	47	40	40	45	43	43	46	42	46	43	46	41	33	41	44	46	38
PPII$_{(Eq2)}$	46	54	58	58	59	56	54	57	51	55	61	58	61	56	57	58	55	53	51	46	50	49	50
E. coli membranes																							
Class	R11	L1	L2	L3	L4	L5	L6	L7	L8	L9	L10	L11	W1	W2	W3	W4	W5	W6	W7	W8	W9	W10	W11
H	3	0	0	0	0	0	0	0	0	1	0	6	1	0	0	1	0	5	0	6	5	5	1
S	24	16	26	17	21	20	22	15	23	24	18	17	20	28	22	30	24	17	19	11	21	11	14
Turn	11	11	11	11	11	11	11	11	12	12	10	13	10	14	12	14	12	16	15	16	16	16	12
PP2	13	16	16	14	15	14	14	15	14	13	17	14	13	15	14	14	14	14	15	14	12	15	13
Unrd	49	57	48	57	52	55	53	59	52	51	55	51	56	43	51	41	50	84	52	53	46	53	60
PPII$_{(Eq2)}$	52	74	73	71	71	64	62	69	62	54	79	71	56	64	58	50	58	45	46	44	42	46	48
DMPG																							
Class	R11	L1	L2	L3	L4	L5	L6	L7	L8	L9	L10	L11	W1	W2	W3	W4	W5	W6	W7	W8	W9	W10	W11
H	0	0	0	0	0	0	0	1	0	0	6	0	3	7	6	36	47	19	16	0	18	0	0
S	21	15	22	19	23	17	19	13	20	23	12	12	25	23	17	0	0	8	2	55	10	38	45
Turn	12	11	10	11	10	11	13	12	10	11	15	11	15	15	15	19	53	26	19	10	22	12	11
PP2	16	16	16	15	16	15	15	15	14	14	14	17	12	10	10	45	0	10	8	20	9	20	14
Unrd	52	58	52	56	51	57	53	60	56	52	53	60	46	46	53	0	0	38	55	16	41	30	29
PPII$_{(Eq2)}$	71	80	79	73	76	73	68	71	65	55	83	89	44	47	36	51	57	51	35	64	46	68	55
